# Joint modeling of longitudinal changes of pulse rate and body temperature with time to recovery of pneumonia patients under treatment: a prospective cohort study

**DOI:** 10.1186/s12879-023-08646-6

**Published:** 2023-10-12

**Authors:** Getu Dessie Biru, Muluwerk Ayele Derebe, Demeke Lakew Workie

**Affiliations:** 1 Department of Statistics, Dembi Dolo University, Debretabor University, Ethiopia; 2 Department of Statistics, College of Science, Bahir Dar University, Bahir Dar, Ethiopia

**Keywords:** Pneumonia, Joint model, Pulse rate, Body temperature

## Abstract

**Background:**

Pneumonia is the leading infectious cause of mortality worldwide and one of the most common lower respiratory tract infections that is contributing significantly to the burden of antibiotic consumption. The study aims to identify the determinants of the progress of pulse rate, body temperature and time to recovery of pneumonia patients.

**Method:**

A prospective cohort study design was used from Felege Hiwot referral hospital on 214 sampled pneumonia patients from March 01, 2022 up to May 31, 2022. The Kaplan–Meier survival estimate and Log-Rank test was used to compare the survival time. Joint model of bivariate longitudinal and time to event model was used to identify factors of longitudinal change of pulse rate and body temperature with time to recovery jointly.

**Result:**

As the follow up time of pneumonia patient’s increase by one hour the average longitudinal change of pulse rate and body temperature were decreased by 0.4236 bpm and 0.0119 ${C}^{0}$. The average longitudinal change of pulse rate and body temperature of patients who lived in rural was 1.4602 bpm and 0.1550 ${C}^{0}$ times less as compared to urban residence. Patients who had dangerous signs are significantly increased the average longitudinal change of pulse rate and body temperature by 2.042 bpm and 0.6031 ${C}^{0}$ as compared to patients who had no dangerous signs. A patient from rural residence was 1.1336 times more likely to experience the event of recovery as compared to urban residence. The estimated values of the association parameter for pulse rate and body temperature were -0.4236 bpm and -0.0119 respectively, which means pulse rate and body temperature were negatively related with patients recovery time.

**Conclusion:**

Pulse rate and body temperature significantly affect the time to the first recovery of pneumonia patients who are receiving treatment. Age, residence, danger sign, comorbidity, baseline symptom and visiting time were the joint determinant factors for the longitudinal change of pulse rate, body temperature and time to recovery of pneumonia patients. The joint model approach provides precise dynamic predictions, widespread information about the disease transitions, and better knowledge of disease etiology.

## Background

Pneumonia is a kind of ARTI (acute respiratory tract infection) that affects the lungs. When a person gets pneumonia, the alveoli in their lungs become clogged with discharge and fluids, making breathing difficult and cutting off oxygen uptake [1]. Pneumonia is an irritation of the lungs including the alveolar pipes and alveolar sacs. It is related to intense respiratory parcel disease and as of late evolved radiological signs. The most common way to classify pneumonia is by where or how it was acquired. Community-acquired pneumonia (CAP) is a type of pneumonia that develops outside of the hospital and is identified within 48 h after admission. Hospital-acquired pneumonia, on the other hand, occurs longer than 48 h after admission and without any prior indications of infection at the time of admission [2].

Pneumonia can be defined as community-acquired, hospital-acquired (nosocomial), or ventilator-associated pneumonia, depending on the location and mode of occurrence. It can also be classified as lobar, bronchial, or acute interstitial pneumonia, depending on the area of the lung affected. It can be classified as non-severe, severe, or very severe based on indications and symptoms. A productive cough, fever with shaking chills, shortness of breath, severe or stabbing chest discomfort during deep breathing, and an accelerated pace of breathing are all [3]. Infection with viruses or bacteria is the most common cause of pneumonia. It can be caused by viruses or bacteria in up to 45 percent of children and 15 percent of adults. The most prevalent cause of community-acquired pneumonia (CAP) is bacteria, with Streptococcus pneumonia being detected in over half of all cases. Haemophilus influenza is found in 20% of cases, Chlamydophila pneumonia is found in 13% of cases, and Mycoplasma pneumonia is found in 3% of cases. Drug-resistant forms of the aforesaid infections, such as drug-resistant Streptococcus pneumonia (DRSP) and methicillin-resistant Staphylococcus aurous, are becoming more common in children, accounting for around 15% of pneumonia cases [4].

The microbiological etiology of health-care-associated pneumonia (HCAP) is more similar to HAP than CAP due to patient risk factors. The difficulties of defining risk indicators for this population, combined with the global variability of post-hospital health care, show that the concept of HCAP is of low utility, and it was left out of recent CAP and HCAP guidelines. Pneumonia also causes pulmonary dysfunction and the start or worsening of cardiovascular disease (CVD); includes heart attacks, strokes, heart failure, and atrial fibrillation), as well as cognitive decline, depression, physical limitations, and a shorter lifespan. Although the disease is caused by a host pathogen interaction, the host's features are the most important predictors of susceptibility to, progression through, and outcomes from pneumonia. Although much attention has been paid to the microorganisms that cause pneumonia, there is still a great need for research from the standpoint of the host [5].

CAP is a truly worldwide disease that continues to be the largest infectious disease cause of hospitalization, morbidity, and death in both developed and developing countries. Based on disparities in population ages and smoking habits, underlying comorbid diseases, influenza and pneumococcal vaccination rates, and access to healthcare, clinical and microbiological differences in CAP exist in different parts of the world. Pathogen epidemiological trends are also shifting in different geographical regions. Streptococcus pneumonia (pneumococcus) has long been thought to be the most common cause of CAP. Pneumococcus is one of the most common causes of CAP in Europe and Sub-Saharan Africa, with continuously high levels of pneumococcal infection after successful antiretroviral medication rollout in Sub-Saharan Africa, owing mostly to underlying human immunodeficiency virus infection [6]. However, longitudinal studies typically have more than one repeated response variables which can be related to time to event outcome. In this study, we would be focused on joint modelling of bivariate longitudinal outcomes and time to-event outcome.

## Materials and methods

### Study area

The study was conducted at Felege Hiwot Referral Hospital (FHRH), Bahir Dar, Ethiopia. The Hospital gives service to people who are living in Northwest Ethiopia. It is away 564 KM from Addis Ababa, the capital city of Ethiopia [7].

### Variables in the study

#### Dependent variables

This study considered three response variables. These are the two longitudinal measures and one event-time outcome. The longitudinal responses of pulse rate and body temperature of pneumonia patients were measured approximately every six hours during the day. Because, Monitoring pulse rate and body temperature as longitudinal variables in pneumonia allows for early detection of infection, assessment of disease severity, evaluation of treatment effectiveness, identification of complications, and prognostic assessment. These vital signs provide valuable information for effective management and monitoring of patients with pneumonia.

*Pulse rate (PR)*: is the number of beats per minute (bpm) measured over one full minute.

*Body temperature (BT)*: is a measure of how well your body can generate, expel heat in degrees Celsius and it is also measured for one full minute.

*Time to event data*: Time to the recovery of diseases in hours.

PR and BT from baseline up to hospital discharge of 214 sample pneumonia patients were measured correspondingly and approximately every six hours of the day and considered a maximum of 17 follow ups for each of the two outcomes. Data were collected at the 6, 12, 18, 24, 30, 36, 42, 48, 54, 60, 66, 72, 78, 84, 90, 96, and 102 h visits, beginning with the baseline and continuing every six hours. The time to recovery of pneumonia patients can be given as:

$$status=\left\{\begin{array}{c}0,if\;not\;recover\\1,\;recover\end{array}\right.$$

### Independent variables

The list of independent variables and their description, categories, and coding were presented in Table 1.

**Table 1:** Independent variables and coding

Variables	Categories(code)
Age of patient	Age ≤ 14 = 0, 15 ≤ Age ≤ 24 = 1, 25 ≤ Age ≤ 64 = 2, Age ≥ 65 = 3
Sex of patient	Female = 0, Male = 1
Residence of the patient	Rural = 1, Urban = 0
Patient use of toilet	Open pit-latrine = 0, ventilated improved pit-latrine = 1
Weekly household income	Income ≤ 500 = 0, 500 ≤ Income ≤ 1500 = 1, Income ≥ 1500 = 2
Patient source of drinking water	Piped = 0, Others = 1
Number of household members	Member ≤ 4 = 0, Member > 4 = 1
Baseline symptoms	Cough = 0, Fast breathing = 1, Others = 2
Presence of comorbidity	No = 0, yes = 1
presence of dangerous signs	No = 0,yes = 1
Patient occupation	Unemployed = 0, employed = 1
Patient visiting time	Follow up time in hours

### Statistical methods

The data were explored using individual profile plots, mean profile plots, and the Kaplan–Meier curve. Then, the longitudinal measures from PR and BT were taken within follow-up, and the time to recovery of diseases in hours of patients was analysed separately to identify the determinant factors for both models, and jointly to assess the influence of the longitudinal change of PR and BT on survival time till to the first recovery among pneumonia patients via linear mixed effects model (LMM).

### Joint model of univariate longitudinal and survival data

The structure of the joint modelling requires a model of longitudinal response and time to event data. These two responses should be modelled simultaneously; therefore, a structure for.

considering the association between them is required [8]. Besides, for the vector of fixed effects of the longitudinal sub-model by assuming individuals with different levels of pulse rate and temperature variability have different susceptibility to recovery. Additionally, the pulse rate and body temperature trajectory is described by the LMM that incorporates subject specific variance [9]. Thus, the longitudinal sub model given as:

$$\begin{array}{c}{\mathrm{y}}_{\mathrm{i}}\left(\mathrm{t}\right)={{\mathrm{x}}^{\mathrm{^{\prime}}}}_{\mathrm{i}}\left(\mathrm{t}\right)\upbeta +{{\mathrm{z}}^{\mathrm{^{\prime}}}}_{\mathrm{i}}\left(\mathrm{t}\right){\mathrm{b}}_{\mathrm{i}}+{\upvarepsilon }_{\mathrm{i}}\\ ={\mathrm{w}}_{\mathrm{i}}\left(\mathrm{t}\right)+{\upvarepsilon }_{\mathrm{i}}\left(\mathrm{t}\right), {\mathrm{w}}_{\mathrm{i}}\left(\mathrm{t}\right)={{\mathrm{x}}^{\mathrm{^{\prime}}}}_{\mathrm{i}}\left(\mathrm{t}\right)\upbeta +{{\mathrm{z}}^{\mathrm{^{\prime}}}}_{\mathrm{i}}(\mathrm{t}){\mathrm{b}}_{\mathrm{i}}\end{array}$$

The parameters ${\mathrm{b}}_{\mathrm{i}}\sim \mathrm{N}\left(0,\mathrm{G}\right), {\upvarepsilon }_{\mathrm{i}}(\mathrm{t})\sim \mathrm{N}(0, {{\updelta }_{\mathrm{I}}}^{2})$ where, ${\mathrm x}_{\mathrm i}\left(\mathrm t\right)\;\mathrm{and}\;z_{\mathrm i}(\mathrm t)$ are the design vectors for fixed effect $\upbeta$, and ${\mathrm{b}}_{\mathrm{i}}$ is a vector of random slope effects of ${\mathrm{Z}}_{\mathrm{i}}$ and ${\upvarepsilon }_{\mathrm{i}}\left(\mathrm{t}\right)$ are the error terms. The random effects ${\mathrm{b}}_{\mathrm{i}}$ follow a bivariate normal distribution with covariance matrix $\mathrm{G}$ which means the Random effects account for individual-level differences or heterogeneity that may exist among the subjects in a longitudinal study. The error terms are normally distributed, and independent of ${\mathrm{b}}_{\mathrm{i}}$. To handle the measurement error, the observed longitudinal outcome ${\mathrm{y}}_{\mathrm{i}}(\mathrm{t})$ is expressed as the sum of the true longitudinal outcomes ${\mathrm{w}}_{\mathrm{i}}(\mathrm{t})$ and a random error term in the mixed effects model [10, 11].

The goodness of fit of the joint longitudinal survival model was checked using the novel decomposition of AIC and BIC [12].

### Bivariate longitudinal sub model

The longitudinal sub model is a linear mixed model (LMM) for a continuous covariate, or a generalized LMM for a discrete covariate (e.g. count, binary variable). This study focused on the LMM assuming homogeneous within patient variance. Let ${\mathrm{y}}_{\mathrm{k}}({\mathrm{t}}_{\mathrm{ij}})$ denote the observed measurements of the ${\mathrm{k}}^{\mathrm{th}}$ longitudinal outcome for subject i at time points${\mathrm{t}}_{\mathrm{ij}}$, where ${\mathrm{j}=\mathrm{1,2},3,..\mathrm{ n}}_{\mathrm{i}}$, $\mathrm{i}=\mathrm{1,2},3,\dots \mathrm{n}$ $\mathrm{k}=\mathrm{1,2}$ denotes the number of longitudinal outcomes; ${\mathrm{n}}_{\mathrm{i}}$ is the number of longitudinal repeated measures for each outcome; k is the number of longitudinal outcomes in the model [13]. The model we describe is the natural extension of the model proposed by [14] to the case of multivariate longitudinal data. The model posits an unobserved or latent zero-mean *(K* + 1*)* variation Gaussian process that is realized independently for each subject,

$${\mathrm{w}}_{\mathrm{i}}\left(\mathrm{t}\right)=\left[{{\mathrm{w}}_{1\mathrm{i}}}^{1}\left(\mathrm{t}\right),..{{\mathrm{w}}_{1\mathrm{i}}}^{\mathrm{k}}(\mathrm{t}){\mathrm{w}}_{\mathrm{i}}(\mathrm{t})\right]$$

This latent process subsequently links the separate sub-models via association parameters.

The ${\mathrm{k}}^{\mathrm{th}}$ longitudinal data sub-model is given by

$$\begin{array}{c}{\mathrm{y}}_{\mathrm{ik}}\left({\mathrm{t}}_{\mathrm{i}}\right)={\upmu }_{\mathrm{ik}}\left({\mathrm{t}}_{\mathrm{i}}\right)+{w}_{ik}\left(\mathrm{t}\right)+{\upvarepsilon }_{\mathrm{ik}}\left(\mathrm{t}\right) \qquad {\upvarepsilon }_{\mathrm{ik }}\sim {\varvec{N}}\left(0,{{\varvec{\sigma}}}_{{\varvec{k}}}^{2}\right)\boldsymbol{ }and\ \mathrm{k}=2,\text{ which is PR and BT}\\ {\upmu }_{\mathrm{ik}}\left({\mathrm{t}}_{\mathrm{i}}\right)={{\mathrm{x}}_{\mathrm{ik}}}^{\mathrm{T}}{\upbeta }_{\mathrm{k}}\ \text{is mean of }{\mathrm{k}}_{\mathrm{i}} \\ \mathrm{and}\qquad {w}_{ik}={{\mathrm{Z}}_{\mathrm{ik}}}^{\mathrm{T}}{\mathrm{b}}_{\mathrm{ik}}\end{array}$$

Where, ${{\mathrm{Z}}_{\mathrm{ik}}}^{\mathrm{T}}$ is an ${\mathrm{r}}_{\mathrm{k}}$ vector of possibly covariates with corresponding subject-and-outcome random effect terms ${\mathrm{b}}_{\mathrm{ik}}$ is a matrix of random slope effects of ${\mathrm{Z}}_{\mathrm{ik}}$, which follow a zero-mean multivariate normal distribution with ${(\mathrm{ r}}_{\mathrm{k}}\times {\mathrm{r}}_{\mathrm{k}})$ variance covariance matrix ${\mathrm{D}}_{\mathrm{kk}}$. To account for dependence between the different longitudinal outcomes, individual heterogeneity, models the correlation among repeated measures, captures unobserved factors, and improves the precision and efficiency of parameter estimation, we let ${\mathbf{c}\mathbf{o}\mathbf{v}(\mathrm{b}}_{\mathrm{ik}} ,{\mathrm{b}}_{\mathrm{in}}){=\mathrm{ b}}_{\mathrm{kn}}$ for *k* ≠ *n*. Furthermore, we assume ${\upvarepsilon }_{\mathrm{ik}}$ and ${\mathrm{b}}_{\mathrm{ik}}$ are un-correlated, and that the censoring times are independent of the random effects [10].

### Survival sub model

Assuming that the hazard function depends on some functions of the true longitudinal measures $\mathrm{F}({{\mathrm{y}}_{\mathrm{ik}}\left(\mathrm{t}\right)}^{*}$ and the baseline covariates ${\mathrm{w}}_{\mathrm{i}}$, the hazard function [13]. Let ${\mathrm{h}}_{0}(\mathrm{t})$ denotes the baseline hazard function, and ${\mathrm{\alpha }}_{\mathrm{k}}$ and γ are coefficients for the function of ${\mathrm{k}}^{\mathrm{th}}$ biomarker and baseline risk factors. The baseline hazard function can be a parametric function or a flexible piecewise constant function. The correlation between the multiple longitudinal biomarkers and time to event outcome is induced by the shared random effects through $\mathrm y_{\mathrm k}^\ast\left({\mathrm t}_{\mathrm i}\right)\mathrm{or}\;{\mathrm b}_{\mathrm{ik}}$ or in the longitudinal and survival models. The function $\mathrm{F}(.)$ can be chosen as different functional forms depending on the interest of the study. If the focus is the association between longitudinal values and event risk, the function can be an identity function. The survival sub model of the Cox proportional hazard model is given by [15, 16].

$${\mathrm{h}}_{\mathrm{i}}\left(\mathrm{t}\right)={\mathrm{h}}_{0}\left(\mathrm{t}\right)\mathrm{exp}({\upgamma }^{\mathrm{T}}{\mathrm{w}}_{\mathrm{i}}+{\sum }_{\mathrm{k}=1}^{2}{\mathrm{\alpha }}_{\mathrm{k}}\mathrm{F}({\mathrm{y}}_{\mathrm{k}}*\left({\mathrm{t}}_{\mathrm{i}}\right)))$$

The basic joint model using shared random effects, closely following [9] consists of a survival sub model where the hazard.

$${\mathrm h}_{\mathrm i}\left(\mathrm t\right)={\mathrm h}_0\left(\mathrm t\right)\exp\left(\mathrm x_{\mathrm i}^{\mathrm T}\mathrm\beta+{\mathrm\alpha}_{\mathrm k}{\mathrm w}_{\mathrm i}\left(\mathrm t\right)\right)$$

The model contains the baseline survival covariates and the true longitudinal marker ${\mathrm{w}}_{\mathrm{i}}(\mathrm{t})$ where ${\mathrm{\alpha }}_{\mathrm{k}}$ denotes the association between the longitudinal and the time-to-event process.

There are three common association structures; “current value”, “current value and slope”, and “shared random effects” parameterization [15, 17, 18]. Among those association parameterizations the “current value and slope” was used, hence our interest is to see the effects of the current true values and slopes of Pulse rate (PR) and Body temperature (BT) on Time to the recovery of diseases of Pneumonia patients.

## Results

Data exploration was done using tabular and graphical approaches. Among 214 pneumonia patients, 183(85.51%) were recovered, and 104 (48.6%) were residents from rural areas (see Table 2). About 103(48.1%), 110(51.4%), 91(42.5%), and 130(60.7%) had breathing difficulty, no danger sign, mild-sever and comorbidity respectively.

**Table 2:** Frequency distribution for baseline independent variables together with recovery time difference and their association

	variable	categories	Not-recover	recover	Total	${X}^{2} (p-value)$
1.	sex	male	21(18.6%)	92(81.42%)	113(52.8%)	68.35(< 0.001)
		female	10(9.9%)	91(90.1%)	101(47.2%)	
2	age	$\le$ 14 = children	11(11.6%)	84(88.42%)	95(44.4%)	200(< 0.001)
		14–24 = youth	11(30.6%)	25(69.44%)	36(16.8%)	
		25–64 = adult	3(7.3%)	38(92.68%)	41(19.2%)	
		$\ge$ 65 = senior	6(14.3%)	36(85.72%)	42(19.6%)	
3	residence	rural	11(10.58%)	93(89.42%)	104(48.6%)	67.31(< 0.001)
		urban	20(18.2%)	90(81.82%)	110(51.4%)	
4	Toilet use	Open pit-latrine	20(15.87%)	106(84.13%)	126(58.9%)	9.19(0.0024)
		Ventilated improved	11(12.50%)	77(87.50%)	88(41.1%)	
5	Drinking water	Others	17(13.60%)	108(86.40%)	125(58.4%)	20.28(< 0.001)
		Piped	14(15.73%)	75(84.27%)	89(41.6%)	
6	Baseline symptom	Cough	12(27.27%)	32(72.73%)	44(20.6%)	6.10(0.10)
		Breath difficult	9(8.74%)	94(91.26%)	103(48.1%)	
		Others	10(14.93%)	57(85.07%)	67(31.3%)	
7	family income	< 500	11(15.71%)	59(84.29%)	70(32.7%)	
		500–1500	5(7.25%)	64(92.75%)	69(32.2%)	
		> 1500	15(20.00%)	60(80.00%)	75(35.0%)	
8	severity	Non-sever	10(20.00%)	40(80.00%)	50(23.4%)	218.49(< 0.001)
		Mild-sever	10(10.995)	81(89.01%)	91(42.5%)	
		Sever	11(15.07%)	62(84.93%)	73(34.1%)	
9	Danger sign	No	18(16.36%)	92(83.645)	110(51.4%)	150.01(< 0.001)
		Yes	13(12.50%)	91(87.50%)	104(48.6%)	
10	Family size	$\le$ 4	20(14.81%)	115(85.19%)	135(63.1%)	2.64(0.1043)
		> 4	11(13.92%)	68(86.08%)	79(36.9%)	
11	Occupation status	Unemployed	7(7.53%)	86(92.475)	93(43.5%)	0.9(0.30)
		Employed	24(19.83%)	97(80.17%)	121(56.5%)	
12	comorbidity	No	15(17.86%)	69(82.145%)	84(39.3%)	96.11(< 0.001)
		Yes	16(12.31%)	114(87.69%)	130(60.7%)	
13	Patient Status		Not-recover	31(14.49%)	214(100%)	
			recover	183(85.51%)		

First categories of the variables were assumed to be reference group

The mean and median survival times of pneumonia patients were 63.34 and 66 h, respectively. The mean PR and BT values were 107.04 bpm and 37.26 0C, with standard deviations of 23.83 and 0.7884, respectively.

**Fig. 1:**
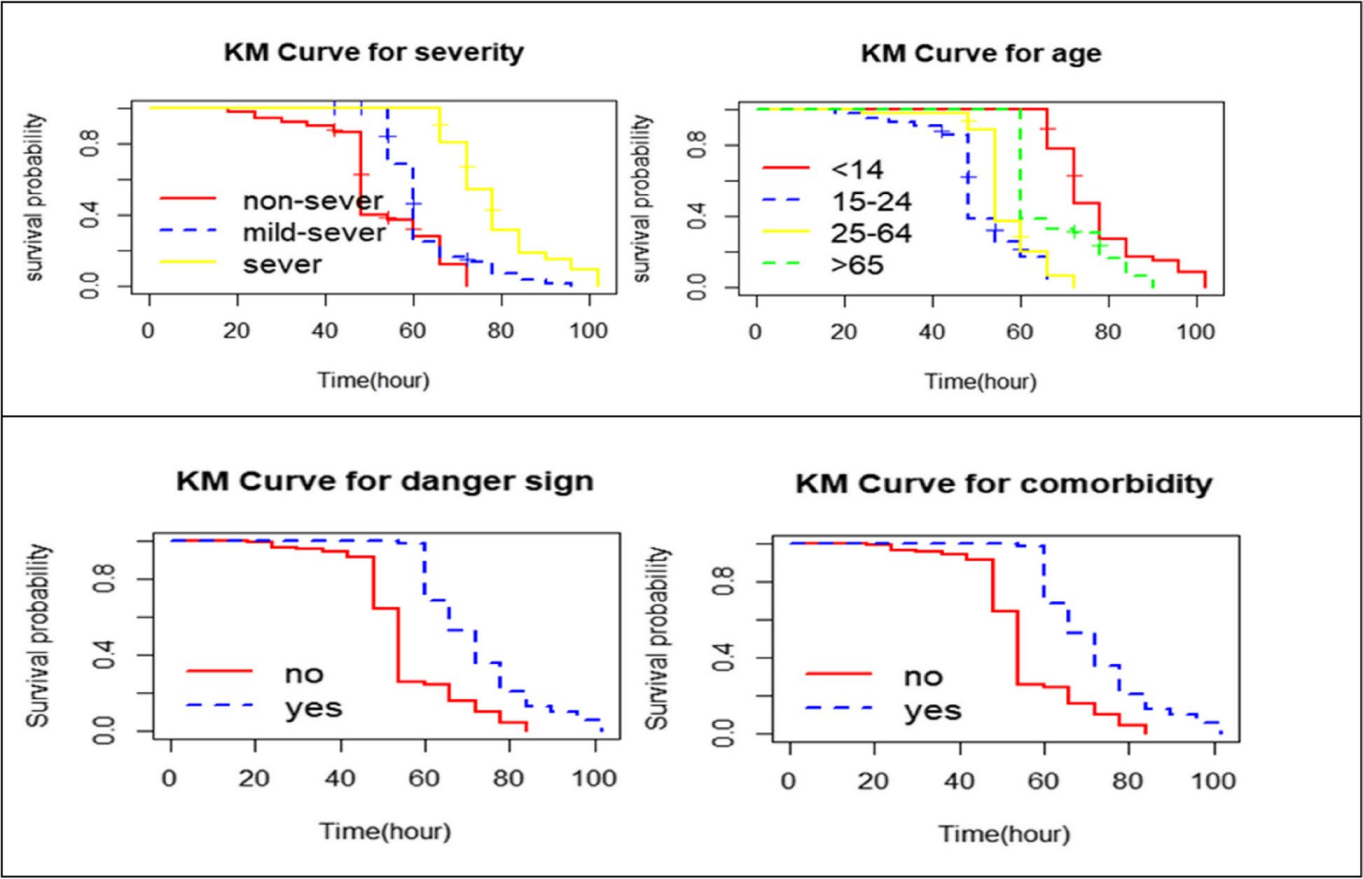
The Kaplan–Meier survival curves for severity, age, danger sign and comorbidity

The Kaplan–Meier survival curves in Fig. 1 revealed that the recovery time of pneumonia patients having comorbidity required longer time to recover from pneumonia as compared to the recovery time of patients without comorbidity.

**Fig. 2:**
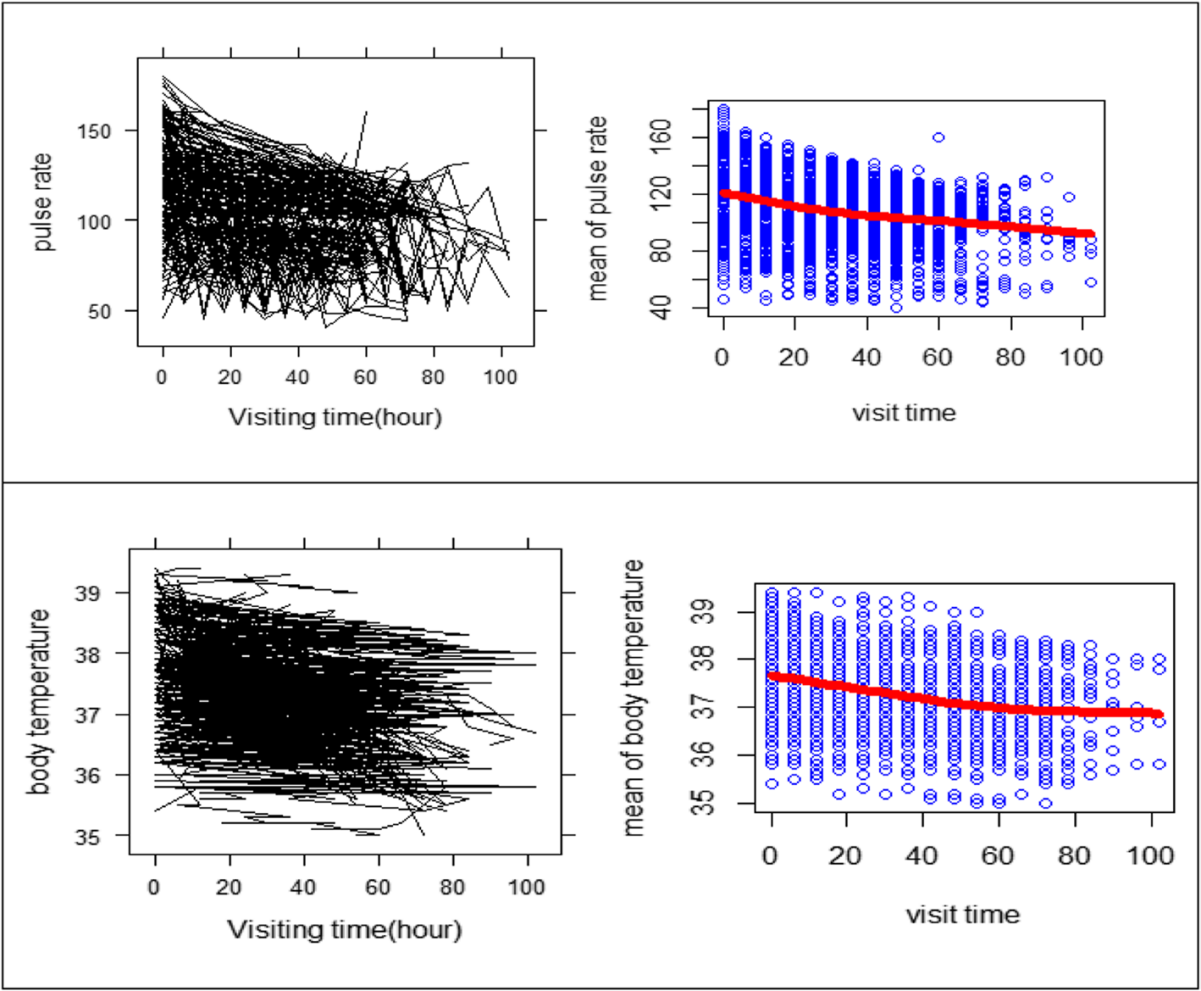
Individual profiles with average trend line for PR and BT

The profile plot in Fig. 2 depicts that the patients began with a varying baseline of PR and BT. It also shows the progress of PR and BT are different over time. The mean profile shows patients' PR stays relatively constant during the research period, while their BT declines with time.

**Fig. 3:**
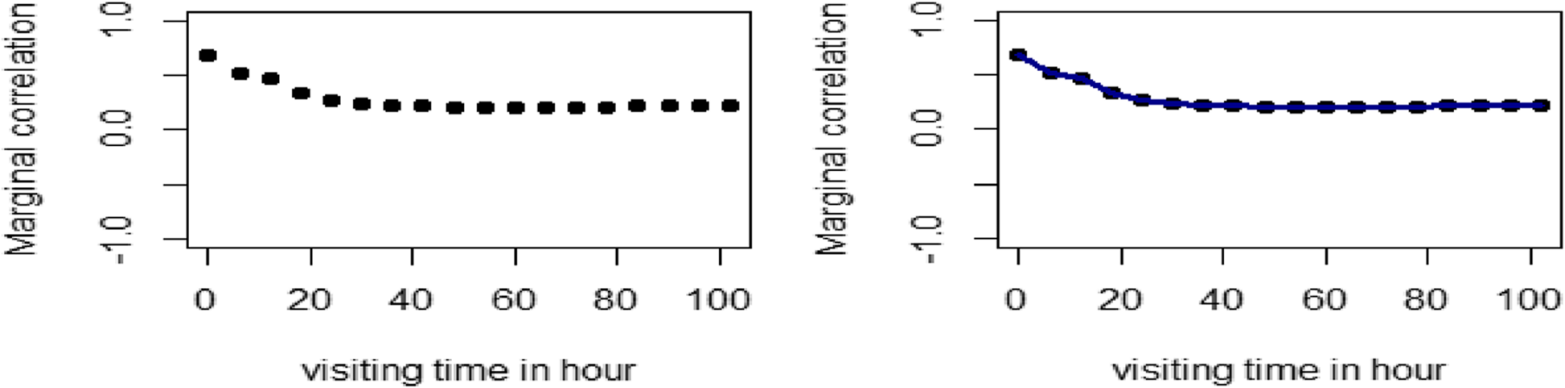
Marginal correlation plot for PR and BT of pneumonia patients

The evolution of association was used to find the marginal correlation between the two responses PR and BT at different visit times. i.e., for the first two visit times, the marginal correlation between the two measures was 0.0.6846 (at the first visit), and 0.5198 (at the second visit, which shows a little decrement (see Fig. 3).

The estimated standard deviation of random intercepts of PR and BT was -0.4268 (*p*-value < 0.001) and -0.2931(*p*-value < 0.001) respectively, and this shows the average longitudinal change of PR and BT had an association with time to recovery of pneumonia patients. The output result indicates that PR and BT were negatively associated with time to recovery of pneumonia patients. It was providing that the bivariate longitudinal PR and BT were negatively associated with the time to recovery of pneumonia patient who had the treatment follow-up.

From the random part of the model Table 3 shows that the variation of the random intercepts in PR was 228.94 with random slopes of 0.834 and variation of the random intercepts in BT was 0.9567 with random slopes of 0.376. This implies there is higher base line difference in PR and BT in the start of their treatment. The covariance of random intercept and the random slope of the two biomarkers PR and BT were -8.96 and -0.438 respectively. The within variance of the joint model were 13.4975 and 0.4963 for pulse rate and body temperature respectively. Table 3, below displays the result of bivariate longitudinal measures of PR and BT with time to recovery of pneumonia patients modelling jointly.

**Table 3:** Joint model parameter estimates for longitudinal and survival processes

**Multivariate linear mixed-effects sub-model**						
	**Pulse rate**			**Body temperature**		
**Variables**	$\widehat{{\varvec{\beta}}}(95\boldsymbol{\%}\boldsymbol{ }{\varvec{C}}{\varvec{I}})$	***Se(*** $\widehat{{\varvec{\beta}}}$***)***	***p*-value**	$\widehat{{\varvec{\beta}}}(95\boldsymbol{\%}\boldsymbol{ }{\varvec{C}}{\varvec{I}})$	***Se(*** $\widehat{{\varvec{\beta}}}$***)***	***p*-value**
**Intercept**	117.92(112.021, 123.829)	3.0123	0.0001	38.2809(38.0379, 38.5237)	0.1239	0.0001
**Sex**						
**Male**	1.998 (-0.0915, 4.0886)	1.0664	0.0609	0.3675 (0.2849, 0.450016)	0.0421	0.3822
**Age**						
**15–24**	-3.9482(-5.8037, -2.0927)	0.9467	< 0.001	-1.3351(-1.4706, -1.19961)	0.0691	< 0.001
**25–64**	-3.6249(-5.1310, -2.1188)	0.7684	< 0.001	-1.5779(-1.7307, -1.4250)	0.0780	< 0.001
$\ge$ **65**	-2.4263(-4.1840, -0.6686)	0.8968	0.0068	-0.8020(-0.9674, -0.6365)	0.0844	< 0.001
**Resid rural**	-1.4602(-2.3885, -0.5319)	0.4736	0.0021	-0.1550(-0.24359, -0.0664)	0.0436	0.0003
**Dsign yes**	2.0421(0.009, 4.074)	1.0368	0.0489	0.6031(0.4481, 0.7579)	0.0790	< 0.001
**Income**						
**500–1500**	-1.8654(-2.6433, -1.0875)	0.3969	< 0.001	-0.0798(-0.8836, 0.0059)	0.0437	0.0678
**> 1500**	-1.7802(-2.1924, -1.3680)	0.2103	< 0.001	-0.0628(-0.1453, 0.0197)	0.0421	0.1357
**Comorbid yes**	1.2458(0.4308, 2.0608)	0.4158	< 0.001	0.1260(0.0280, 0.2240)	0.0500	0.0117
**Severity**						
**Mild-sever**	1.8298(0.2134, 3.4462)	0.8247	0.0265	0.0816(-0.0069, 0.1702)	0.0452	0.0710
**Sever**	2.1463(0.1228, 4.1698)	1.0324	0.0376	0.0853(-0.0080, 0.1786)	0.0476	0.0731
**Toilet**						
**Ventilated**	-1.2843(-2.6822, 0.1136)	0.7132	0.0717	-0.4186(-0.6354, -0.2018)	0.1106	< 0.001
**Water Other**	1.5264(-0.0071, 3.0599)	0.7824	0.0510	0.1014(-0.0797, 0.2825)	0.0924	0.2724
**Baseline sign**						
**Breath**	1.4021(0.2010, 2.6032)	0.6128	0.0221	0.5128(0.0949, 0.9307)	0.2132	0.0161
**Others**	1.5192(0.2212, 2.8173)	0.6623	0.0218	0.4678(0.1448, 0.7908)	0.1648	0.0045
**Occupation**						
**Employee**	1.0043(-0.2546, 2.2636)	0.6425	0.1180	0.4265(-0.1111, 0.9641)	0.2743	0.1199
**Family size > 4**	0.7264(-0.0227, 1.4755)	0.3822	0.0573	0.6178(-0.2728, 1.5084)	0.4544	0.1739
**Visit time**	-0.4236(-0.4572, -0.3900)	0.0171	0.0001	-0.0119(-0.0131, -0.0105)	0.0007	< 0.001
**Random effects of variance covariance matrix**						
		**PR**		**BT**		
		${\boldsymbol{\alpha }}_{{\varvec{i}}1}$	${{\varvec{b}}}_{{\varvec{i}}1}$	${\boldsymbol{\alpha }}_{{\varvec{i}}2}$	${{\varvec{b}}}_{{\varvec{i}}2}$	
PR	${\alpha }_{i1}$	228.94	-8.96	12.852	-0.984	
	${b}_{i1}$	-8.96	0.834	-0.186	0.246	
BT	${\alpha }_{i2}$	12.852	-0.186	0.9567	-0.438	
	${b}_{i2}$	-0.984	0.246	-0.438	0.376	
Standard deviation		15.131	0.3851	0.6326	0.0117	
${\varepsilon }_{i1}$		13.4975				
${\varepsilon }_{i2}$		0.4963				
**Survival sub model**						
**Variable parameter**		$\widehat{\beta }(95\% CI$	*Se(* $\widehat{\beta }$*)*	HR	*p*-value	
**Sex reference = **	**Female/Male**	-1.1859(-2.8096, 0.4378)	0.8284	0.3055	0.1522	
**Age**	**15–24**	0.9819(0.1758, 1.7881)	0.4113	2.6695	0.0169	
**ref = **	$\le$ **14/25–64**	0.8679(0.0512, 1.6846)	0.4167	2.3819	0.0372	
	$\ge$ **65**	-0.1043(-0.2058, -0.0028)	0.0518	0.9010	0.0441	
**Weekly household income**	**500–1500**	0.3623(-0.0575, 0.7821)	0.2142	1.2999	0.0907	
**Ref = **	** < 500/ > 1500**	0.6364(-0.0314, 1.3042)	0.3407	2.4508	0.0617	
**Toilet use ref = **	**Open/Ventilated**	-0.6534(-1.4797,0.1729)	0.4216	1.9220	0.1211	
**Danger sign ref = **	**No/Yes**	-1.0626(-1.5052, -0.6200)	0.2258	0.3456	< 0.001	
**Residence ref = **	**Urban/Rural**	0.1254(0.0055, 0.2454)	0.0612	1.1336	0.0404	
**Drinking water ref = **	**Piped/Others**	-0.2516(-0.4948, -0.0084)	0.1241	0.8136	0.7571	
**Comorbidity ref = **	**No/Yes**	-0.8312(-0.8447, -0.8177)	0.0069	0.4355	< 0.001	
**Occupation**	**Unemployed**	-0.2213(-1.4532, 1.0106)	0.6285	0.8015	0.7247	
**Family member**	**> 4**	-0.6648(-1.4123, 0.0827)	0.3814	0.5144	0.0813	
**Severity**	**Mild-Sever**	-0.3874(-0.5469, -0.2279)	0.0814	0.6788	< 0.001	
**ref = **	**Non-sever/Sever**	-0.2612(-0.3643, -0.1581)	0.0526	0.5060	< 0.001	
**Baseline symptom**	**Breathing**	-0.9037(-1.8145, 0.0071)	0.4647	0.4051	0.0518	
**Ref = **	**Cough/Others**	-0.7462(-1.5820, 0.0886)	0.4259	0.4742	0.0797	
${\rho b}_{PR}$		-0.4268 (-0.7428, -0.1108)	0.1612	0.6526	0.0081	
${\rho b}_{BT}$		-0.2931 (-0.5162, -0.0701)	0.1138	0.7459	0.0100	

Key:${\rho b}_{PR}$ the association parameter for slope of PR, and ${\rho b}_{BT}$ the association parameter for the slope of BT respectively

The estimated intercept value of average PR and BT of pneumonia patients were 117.92 bpm and 38.2809 ${C}^{0}$ respectively when the covariates are at the reference category. As the patient’s vising time increase by one hour the average PR and BT of the patient will be decreased by -0.4236 bpm and -0.0119 ${C}^{0}$ respectively (see Table 3). The average PR and BT of patients had a 3.9482 bpm and 1.335 ${C}^{0}$ decrement for patients whose age 15–24 years old than those patients whose age $\le 14$ years old, the average PR and BT of pneumonia patients had a 3.6249 bpm and 1.577 ${C}^{0}$ decrement for patients whose age 25–64 years old than those patients whose age $\le 14$ years old respectively, and the average PR and BT of pneumonia patients had a 2.4263 bpm and 0.802 ${C}^{0}$ decrement for patients whose age $\ge 65$ years old than those patients whose age $\le 14$ years old respectively. Similarly, the average PR and BT of patients had a 1.4602 bpm and 0.1550 ${C}^{0}$ decrements for patients who lived in rural residence than patients who lived in urban residence respectively. The expected change of PR and BT patients had a 2.042 bpm and 0.6031 ${C}^{0}$ increase for patients who had dangerous signs than patients who had no dangerous signs. And also, the average PR and BT of patients had a 1.2458 bpm and 0.1260 ${C}^{0}$ increments for patients who had comorbidity than had no comorbidity diseases. Additionally, the average PR and BT of patients had a 1.2843 bpm and 0.4186 ${C}^{0}$ decrement patients who had ventilated improved pit-latrine toilet than patients who had open pit-latrine toilet in the yard. Finally, the average PR and BT of patients had 1.4021 bpm, 1.5192 bpm and 0.5128 ${C}^{0}$, 0.4678 ${C}^{0}$ increment patients who had baseline symptoms and others than patients who had baseline cough symptom respectively, keeping the other covariates constant.

The hazard of the patient’s time to recovery for aged 15–24 and 25–65 years was 2.6695 and 2.3819 times more likely than the hazard of pneumonia patient’s whose age less than 14 years old respectively, and patent’s aged greater than 65 years was 0.9010 times less likely than patient’s aged less than 14 years (see Table 3). And, the hazard of the patients time to recovery for patients who had dangerous signs was 0.3456 time less likely than patients who had no dangerous signs. Similarly, the hazard of the patient’s time to recovery for patients who lived in rural was 1.1336 times more likely than the hazard of pneumonia patients who lived in urban. Finally, the hazard of the patients time to recovery for patients who had comorbidity diseases was 0.8312 time less likely than patients who had no comorbidity diseases, keeping the other covariates constant.

The time to recovery for patients who had a mild-sever status of pneumonia patients was 68% lower than those who had non-sever pneumonia patients, and the time to recovery for patients who had a sever status of pneumonia patients was 49% lower than those who had non-sever pneumonia patients.

The estimate of the association parameter for the current true value of PR (${\rho b}_{PR}$) in the joint model was -0.4268 (HR = 0.6526 (0.4758 to 0.8951), *p*-value = 0.0081); there is a 0.65-fold decrease in risk of the time to recovery, per doubling of PR. i.e., for a unit bpm increase on the PR the rate of time to recovery of patients will be decreased by 34.74%.

## Discussion

This study attempted to jointly model the longitudinal change of Plus Rate and Body temperature with recovery time of Pneumonia patients receiving treatment. The data were explored using several approaches such as mean plot, profile plot, and Kaplan–Meier estimates. To estimate the effects of the socioeconomic, demographic and biological characteristics joint longitudinal and survival models were employed.

The result reveals that about 85.51% of patients were recovered from pneumonia with a median recovery time of 63.34 h which took shorter recovery time as compared to results of the study done by [19–24], whereas it is longer recovery time as compared to results of the study done by [25–27]. The difference can be due to the difference in explanatory variables that we used and type of hospital etc.

Age has a significant effect on the two longitudinal measures of pneumonia PR and BT. When the age of pneumonia patient’s increase, the average longitudinal change of PR and BT measures are decrease. This indicates that, lower levels of pneumonia are found for increased age of patients. This was in line with results of the study conducted using nonlinear mixed model by [22].

Age has a significant effect on the recovery time of pneumonia patients in this study. That means at age $\le 14$ and $\ge 65$ year age group the recovery time of pneumonia patient is decrease. And at 15–24 and 25–64 age groups the recovery time of pneumonia is increase based on the above output table 4.10. This study confirms the study done on [28–30]. Unlikely, using binomial logistic regression [31] found that age had no significant effect on measures of pneumonia. This requires further investigation to reach a decision in the effects of age on CAP.

The recovery rate of pneumonia patients who have comorbidity, sever pneumonia and age greater than or equal to 65 are decreased as compared to without comorbidity, non-sever pneumonia, age less than or equal to 14 year. This finding is in line with other study done by [28]. And rural residency significantly decreases the average longitudinal change of PR and BT of pneumonia patients. This indicates that, rural residency was significantly associated with the recovery time of pneumonia patient’s. This was in line with the results of the study done by [24, 31].

Urban residency was significantly decreases the recovery time of pneumonia patients where, rural residency were significantly increases the recovery time of pneumonia patients. This finding is confirms the study done on [31]. And the patients who uses ventilated improved pit-latrine toilet were significantly increases the recovery time of pneumonia patients as compared to use open pit-latrine toilet. This finding confirms with the other study done [22].

As the visiting time increases, the average values of pulse rate and body temperature decreases through visit time of pneumonia patients, which indicates the effectiveness of the treatment to lower pneumonia. Visiting time of pneumonia patient had negative association with the average longitudinal change of pulse rate and body temperature of pneumonia patient. This result also conforms from the previous result done by [32].

The age of patient had statistically negative significant effect on average longitudinal change of pulse rate, body temperature and associated with a risk recovery of patient as the age increase the survival of patient increase. This finding is in line with other study done by [33, 34].

Higher values of pulse rate and body temperature were related with longer recovery time (high risk of pneumonia) and low values of pulse rate and body temperature related with shorter recovery time (low risk of pneumonia). This indicates there is association between pulse rate and body temperature with time to recovery of pneumonia patients. This was consistent with results of the studies done by [24, 26, 27].

## Conclusion

The study investigated and identified factors that are associated with bivariate longitudinal measure of PR and BT with time to recovery of pneumonia patient’s treatment at FHSH using joint model of bivariate longitudinal and survival analysis. From the log-rank tests showed that the survival experience of different groups of pneumonia patients on residence, age, danger sign, baseline severity, weekly household income and comorbidity were statistically significant. The bivariate longitudinal linear mixed effect model shows that the correlation between two biomarkers PR and BT of pneumonia had correlation overtime.

From separate analysis the significant factors of pulse rate were age, residence, dangerous sign, baseline severity, weekly household income, comorbidity, and toilet use, baseline symptom and visiting time. And Residence, age, dangerous sign, baseline severity, comorbidity, toilet use, baseline symptom and visiting time are statistically significant effect on average change of body temperature. Whereas age, baseline severity, dangerous sign, toilet use, source of drinking water, residence, comorbidity and weekly household income are statistically significant effect on recovery time of pneumonia patients.

The joint model result indicates that, the significant factors of pulse rate and body temperatures of pneumonia patients were age, comorbidity of diseases, dangerous signs and baseline symptoms whereas risk factors for time to recovery of pneumonia patients were age, residence, dangerous sign, comorbidity, and baseline severity. Weekly household income is a significance predictor of pulse rate of pneumonia patients. And toilet use is a significant variable for body temperature of pneumonia patients. Whereas baseline severity is a significant predictor for both pulse rate and time to recovery of pneumonia patients.

When evaluating the overall performance of both the separate and joint models in terms of model parsimonious, goodness of fit, and the statistical significance of the association parameters, the joint model performs better estimate than the separate models. As the result, we concluded that the joint model was preferred for simultaneous analyses of repeated measurement with survival data.

## Data Availability

The data is owned by the Department of Statistics, Bahir Dar University, Bahir Dar, Ethiopia, and is not publicly available due to ethical restrictions and participant confidentiality concerns. But de-identified data can be available by contacting the Department of Statistics via email: muluwerk.ayelederebe@bdu.edu.et, tel: + 251918282110 for the researchers who meet the criteria for access to confidential data.

## Abbreviation

AFT: Accelerated Failure Time
AIC: Akaike Information Criterion
PR: Pulse Rate
BT: Body Temperature
LMM: Linear mixed effects model
ARTI: Acute respiratory tract infection
CAP: Community-acquired pneumonia
DRSP: Drug-resistant Streptococcus pneumonia
HCAP: Health-care-associated pneumonia
CVD: Cardiovascular disease
FHRH: Felege Hiwot Referral Hospital
bpm: Beats per minute
